# Trehalose alleviates nephropathy in focal segmental glomerulosclerosis via the upregulation of the WT-1/EZH2 pathway

**DOI:** 10.3389/fphar.2025.1706617

**Published:** 2025-11-06

**Authors:** Maha Al-Asmakh, Marwa AL Riyami, Miytha Al Marbuii, Shadia Al Sinawi, Suaad Al-Badi, Faisal AL Ismaili, Amira AlKharusi, Razan Zadjali, Zaina Alharthi, Sara Al Mashrafi, Razan Al Breiki, Sulaiman Al-Hashmi, Ali Al Lawati, Amal Kassab, Fahad Zadjali

**Affiliations:** 1 Department of Biomedical Sciences, College of Health Sciences, QU-Health, Qatar University, Doha, Qatar; 2 Biomedical Research Center, Qatar University, Doha, Qatar; 3 Department of Pathology, College of Medicine and Health Sciences, Sultan Qaboos University, Muscat, Oman; 4 Department of Clinical Biochemistry, College of Medicine and Health Sciences, Sultan Qaboos University, Muscat, Oman; 5 Department of Medicine, The Royal Hospital, Muscat, Oman; 6 Department of Clinical Physiology, College of Medicine and Health Sciences, Sultan Qaboos University, Muscat, Oman; 7 Oman College of Health Sciences, Muscat, Oman; 8 Laboratory for Stem Cell and Regenerative Medicine, Natural and Medical Sciences Research Center, University of Nizwa, Nizwa, Oman; 9 Department of Medicine, Sultan Qaboos University Hospital, Sultan Qaboos University, Muscat, Oman; 10 Biomedical Technology and Cell Therapy Research Laboratory, Department of Biomedical Engineering, Faculty of Medicine, McGill University, Montreal, QC, Canada

**Keywords:** focal segmental glomerular sclerosis, podocyte, fibrosis, renal disease, nephrotic syndrome

## Abstract

**Introduction:**

Focal segmental glomerulosclerosis (FSGS) is a serious disease that culminates in kidney failure. Today, FSGS is diagnosed histologically as a progressive scarring of the glomeruli due to gradual loss or damage of the podocyte layer, making it one of the main targets of FSGS therapeutic approaches. However, given that podocytes are terminally differentiated, post mitotic epithelial cells with limited proliferative capacity, they are considered one of the most vulnerable components of the glomeruli.

**Aim and Methods:**

We herein investigated the effect of trehalose, a naturally occurring sugar with low toxicity and high stability, on kidney function using a murine model of adriamycin‐induced nephropathy.

**Results:**

Based on our data, trehalose administration improved proteinuria in mice with FSGS compared to those without FSGS induction (24 h urine protein of 0.30±0.06 versus 0.55±0.08, p-value<0.05, and urine protein to creatinine ratio of 0.78±0.25 versus 1.56±0.17, p-value<0.05, respectively this is accompanied by reduced fibrosis and podocyte damage. Significant reduction in collagen deposition in glomeruli observed in mice treated with trehalose, P-value<0.01 and significant reduction in glomerular basement membrane thickness, P-value<0.001. Moreover, trehalose intake is associated with higher mature podocyte markers at gene and protein expression levels, Nphs1, Nphs2 and Synpo. These favorable effects seem to be mediated mainly via increased WT-1/EZH2 signaling, which is a key pathway in maintaining normal podocyte function and growth. These effects were also observed in the downstream signaling pathway with lower expression of Mmp-7 and Catenin b1 gene expression (p-value<0.05 and <0.01, respectively).

**Conclusion:**

Our findings suggest that trehalose could be a promising therapeutic agent for FSGS, nevertheless, more studies are necessary to confirm our findings and evaluate trehalose efficacy in clinical settings.

## Introduction

Focal segmental glomerulosclerosis (FSGS) is a chronic kidney disease characterized by progressive scarring of the glomeruli (Jacobs-Cacha et al., 2021). In the absence of less invasive diagnostic markers of the disease, diagnosis of FSGS is based on histopathological examination of a kidney biopsy (Agrawal et al., 2021; Musiala et al., 2022). Although FSGS currently has a low incidence rate of 1.4–21 per million, its diagnosis has been increasing in recent years (Shabaka et al., 2020). More importantly, more than half of FSGS patients gradually progress to end-stage kidney disease within 5–10 years (Zhu et al., 2022), with a recurrence rate in approximately 40% of post-kidney transplant surgery patients (Musiala et al., 2022; Savin et al., 1996). Taken together, FSGS presents one of the biggest medical challenges in kidney disease cases.

The etiology of FSGS remains elusive, with most publications categorizing it into either idiopathic, caused by specific miRNAs and other not yet fully identified circulating permeability factors, or secondary, due to other diseases such as obesity, type 2 diabetes, acquired immune deficiency, viral infections, and drugs, along with genetic factors (Musiala et al., 2022; Savin et al., 1996). Clinical manifestations of the disease are heterogenous, but one staple feature is the varying degrees of proteinuria due to glomerular filtration barrier dysfunction; this is mostly due to damage or loss of podocytes, a hallmark of FSGS (Zhu et al., 2022; Wen et al., 2018). This dysfunction largely results from structural and functional damages to podocytes, whose injury or detachment is considered a hallmark of FSGS pathology. Recent molecular nephrology investigations suggest that alterations in podocyte gene expression, oxidative stress, and dysregulated signaling pathways such as TGF-β, NF-κB, and Notch may also contribute to disease progression and variation in clinical presentation and disease phenotypes (Yuan et al., 2022; Latt et al., 2022).

Podocytes are highly specialized, terminally differentiated, post-mitotic epithelial cells that play a central role in maintaining the integrity of the glomerular filtration barrier (Dai et al., 2009; Yi et al., 2017). They possess a complex cytoskeletal architecture and a network of foot processes, with slit diaphragm structures that maintain selective filtration and mechanical stability. Due to their limited proliferative capacity and inability to replenish their numbers, they are one of the most vulnerable components of the glomeruli. Therefore, understanding podocyte injury and repair mechanisms is crucial for kidney disease treatment and prevention. In addition, podocyte depletion directly correlates with glomerulosclerosis and progressive renal function decline (Dai et al., 2009; Yi et al., 2017).

The primary mechanism of maintaining podocyte homeostasis and promoting their survival is autophagy, which has been shown consistently to be directly linked with kidney health and normal glomerular function (Hartleben et al., 2014; Tang et al., 2019). Under normal conditions, podocyte injury promotes autophagy that inactivates misfolded proteins and organelles while supplying nutrients for survival. Meanwhile, in pathological states, inhibition of autophagy induces apoptosis and aggravates glomerular disease (Yi et al., 2017; Hartleben et al., 2014). It is reported that 20% of podocyte damage is sufficient to induce glomerulosclerosis in mice (Hartleben et al., 2014). As such, autophagy may be considered a protective response to shield podocytes from injury and minimize damage.

The management of FSGS remains a major clinical challenge due to its heterogeneous etiology and variable response to therapy. Current treatment strategies are largely empirical and primarily aimed at reducing proteinuria, preserving renal function, and delaying progression to end-stage kidney disease. Conventional therapy includes tight control of blood pressure to reduce intraglomerular pressure and proteinuria and the use of immunosuppressive therapy (Trachtman, 2020; Caster et al., 2022). Despite these options, a significant proportion of patients fail to achieve sustained remission, highlighting the need for more targeted and pathogenetic treatments. Emerging therapies under clinical investigation include novel immunomodulators, antifibrotic agents, and drugs targeting specific molecular pathways implicated in podocyte injury and permeability factor activity.

In this regard, trehalose, a natural disaccharide of glucose, has demonstrated broad cytoprotective effects in preclinical and clinical research. Animal studies have shown that trehalose enhances autophagy, promotes clearance of misfolded proteins, and reduces oxidative and endoplasmic reticulum (ER) stress (Galbiati et al., 2023; Hosseinpour-Moghaddam et al., 2018) through modulation of pathways such as NRF2, TGF-β, and mTOR, leading to neuroprotection and improved cellular homeostasis in models of neurodegenerative and metabolic diseases (Xu et al., 2023; Zschiedrich et al., 2017; Ruby et al., 2023). It also stabilizes cellular membranes and mitigates inflammation in models of heat stress and oxidative injury (Galbiati et al., 2023; Hosseinpour-Moghaddam et al., 2018). Trehalose has been used in clinical trials to improve tear film stability and symptoms in patients with dry eye disease (Hom et al., 2025), and reduce inflammation markers without affecting glycemic control in patients with type 2 diabetes (van Can et al., 2012). In neurodegenerative disorders, intravenous trehalose administration has been shown to be safe and to slow disease progression (Trial and Group, 2025). Collectively, these findings highlight trehalose as a promising therapeutic candidate that modulates autophagy, reduces oxidative stress, and protects against cellular injury; however, larger controlled clinical trials are still needed to confirm its disease-modifying potential.

More specifically, it has been reported that trehalose can protect podocytes from Adriamycin-induced injury, a well-established experimental model of focal segmental glomerulosclerosis (Kang et al., 2014). *In vitro* studies demonstrated that trehalose treatment significantly reduced podocyte apoptosis and preserved cytoskeletal integrity by stabilizing F-actin organization (Kang et al., 2014). These protective effects are attributed to the promotion of autophagic activity, the attenuation of oxidative stress, and the suppression of proapoptotic signaling pathways, such as caspase-3 and MAPK (Darabi et al., 2018). Nevertheless, to the best of our knowledge, no previous studies have comprehensively investigated the therapeutic potential of trehalose *in vivo* within the context of FSGS. The *in vitro* cellular models fail to fully replicate the complex cellular interactions, hemodynamic forces, and immune mechanisms present in the intact kidney organ. Therefore, *in vivo* investigation is crucial to determine whether the cytoprotective effects observed *in vitro* translate into meaningful structural and functional improvements at the whole-organism level. Therefore, in the present study, we aim to bridge this critical knowledge gap by evaluating the renoprotective role of trehalose as a potential therapeutic agent using an Adriamycin-induced FSGS animal model.

## Materials and methods

### Experimental groups and induction of focal segmental glomerulosclerosis

The study protocol and all experiments were approved by the Ethics Committee for animal use in research at Sultan Qaboos University #SQU/EC-AUR/2021-2022/4.

Forty healthy male BALB/c mice aged 10–12 weeks weighing between 16 and 29 g were randomly allocated to respective experimental groups, with a total of 10 mice per group. Male rodents were used to eliminate hormonal variability and increase reproducibility as estrous cycles in female rodents may alter renal function and affect glomerular injury modulation (Santmyire et al., 2010; Joles et al., 1998). Mice were housed in polypropylene cages with stainless steel covers. Prior experiment, mice were allowed to acclimate to the environment. During the experiment, mice were maintained in a temperature-controlled room (22 C ± 1 C) under standard housing conditions, with a natural 12h light/dark cycle and free access to a standard rodent diet and water.

**FIGURE 1:**
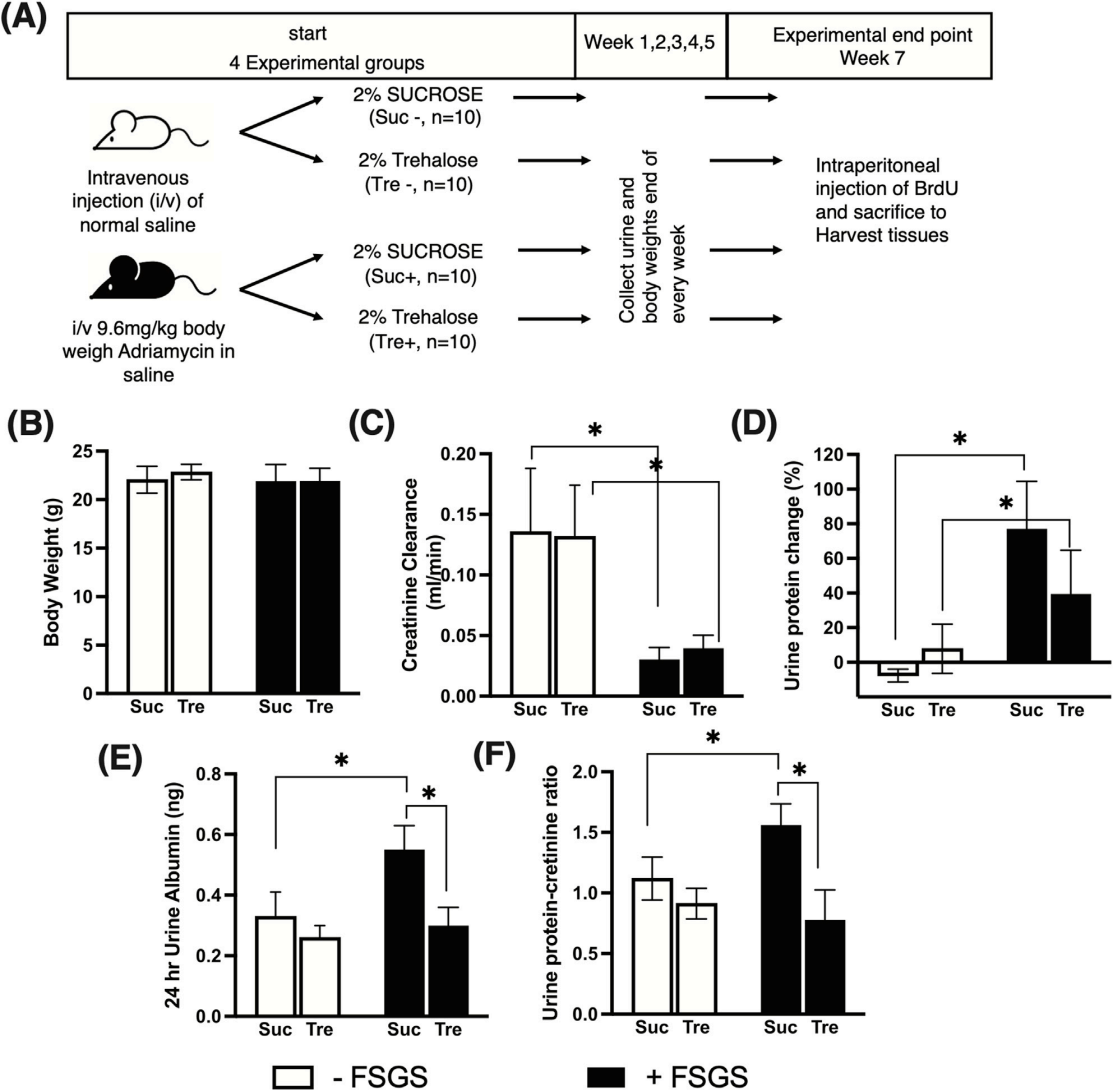
Effect of trehalose administration on markers of subnephrotic proteinuria. **(A)** Experimental design of Adriamycin-induced FSGS. A total of 40 mice were used and divided into four experimental groups (10 mice per group). FSGS was induced (+) via intravenous injection of Adriamycin and controlled using normal saline (−). Post-Adriamycin injection, 2% trehalose (Tre) and sucrose (Suc) were administered in drinking water *ad libitum* for 7 weeks. **(B)** Mouse body weight at the experimental end point. **(C-F)** Markers of kidney function and subnephrotic proteinuria measured at the time of sacrifice. Tukey’s *post hoc* p-values: *<0.05.

The experimental design is described in Figure 1A. To induce FSGS, mice (n = 20) were injected once intravenously with 9.6 mg/kg of Adriamycin (known also as doxorubicin, catalog # ab120629, Abcam, Cambridge, UK) diluted in saline. The selected dose is based on the previous documented use of Adriamycin for FSGS induction (Wyburn et al., 2013; Lee and Harris, 2011). The FSGS-induced group is hereby designated with the “+” sign. The control group with no FSGS induction (n = 20) received saline injection and is designated with the “−” sign. Following injection, mice were administered 2% of either trehalose (Tre) or sucrose (Suc) (catalog # S0389, Sigma-Aldrich, St. Louis, MO, USA) in drinking water for 7 weeks, *ad libitum*. Prior to sacrifice, mice were individually placed in metabolic cages with free access to diet and drinking solution; fresh stool and urine samples were collected every 24 h. Three hours prior to sacrifice, all mice were injected intraperitoneally with bromodeoxyuridine (BrdU, catalog # ab142567, Abcam, Cambridge, UK) to monitor *in vivo* glomerular cell proliferation. Mice were anesthetized using isoflurane (catalog #50019100, Zoetis Inc, Parsippany, NJ, USA), and blood was collected via cardiac puncture. Mice were then scarified by cervical dislocation, and kidneys were harvested and cut into cortical pieces for histochemical analysis and protein and RNA isolation.

### Proteinuria and kidney function assessment

Urine was collected weekly to monitor changes in total protein levels in urine using the Bio-Rad protein assay reagent (catalog # #500-0001, Bio-Rad Laboratories, Hercules, CA, USA). Creatinine was measured in urine and serum using a colorimeter (for urine catalogue # 80350, Crystal Chem, Elk Grove Village, IL, USA, and for serum catalog #KGE005; R&D Systems, Minneapolis, MN, USA). Urine albumin was measured using a mouse-specific ELISA Kit (catalog # 809630, Crystal Chem, Elk Grove Village, IL, USA). The percentage change in urine protein between week 7 and prior to FSGS induction was calculated. Albuminuria was adjusted to the total 24-h urine volume. Creatinine clearance (mL/min) was calculated using the following equation: urine creatinine (mg/dL)/serum creatinine (mg/dL) x urine volume (mL)/collection time (minute). The urine protein-to-creatinine ratio (UPCR) was calculated by dividing urine protein (mg/dL) by the urine creatinine level (mg/dL).

### Histological analysis of glomeruli and podocytes

Dissected kidneys were fixed in 4% formaldehyde overnight at 4 °C and then processed for paraffin embedding. Approximately 3–5 μm-thin sections were stained with hematoxylin and eosin and with Masson’s trichrome for collagen staining. The images of glomeruli were taken under the same light exposure and magnification and then quantified for collagen deposition using Fiji software (https://fiji.sc) (Schindelin et al., 2012; Zadjali et al., 2012). Bromodeoxyuridine (Brdu)-positive cells were identified using anti-Brdu (Clone MoBU-1, Thermo Fisher Scientific, Waltham, MA, USA) at a dilution of 1:50 and then detected using a DAB Ultra-view Universal DAB Detection Kit (catalog # 760-500, Ventana Medical Systems/Roche, Tucson, AZ, USA) and counter-stained with hematoxylin-11 (catalog # 790-2208, Ventana Medical Systems/Roche, Tucson, AZ, USA). The percentage of DAB-stained cells within a glomerulus was determined using the Fiji tool and adjusted to total stained nuclei.

To analyze podocyte integrity, tissue samples were fixed in 2.5% glutaraldehyde for 2 h and then processed for electron microscopy using a JEOL JEM-1230 transmission electron microscope. The Fiji tool was used to measure the glomerular basement membrane (GBM) thickness and foot process width (FPW) (van den Berg et al., 2004).

### Western blot analysis and quantitative real-time PCR

Protein extractions from kidney tissues and SDS-PAGE analysis were performed as previously described (Al-Araimi et al., 2021). Kidney tissues were homogenized in RIPA lysis buffer containing protease and phosphatase inhibitors. Protein concentrations were determined using the Bio-Rad Protein Assay Reagent (Bio-Rad, Hercules, CA, USA). Equal amounts of protein (20–40 µg) were separated using SDS-PAGE and transferred onto PVDF membranes (Millipore, Burlington, MA, USA). Membranes were blocked with 5% non-fat dry milk or BSA in Tris-buffered saline containing 0.1% Tween-20 (TBST) for 1 h at room temperature. Membranes were incubated overnight at 4 °C with primary antibodies at manufacturer-recommended dilutions. After washing with TBST, the membranes were incubated with HRP-conjugated secondary antibodies for 1 h at room temperature (Santa Cruz Biotechnology, Dallas, TX, USA). Protein bands were visualized using the Pierce™ ECL Western Blotting Substrate (Thermo Fisher Scientific, Waltham, MA, USA) and imaged. Band intensities were quantified using Fiji software and normalized to loading controls (β-actin). The following primary antibodies were used: rabbit anti-nephrin (catalog #ab136894, Abcam, Cambridge, UK), mouse anti-Nidgon (catalog #sc-33706, Santa Cruz Biotechnology, Dallas, TX, USA), rabbit anti-Synaptopodin (catalog #ab259976, Abcam, Cambridge, UK), rabbit anti-APG5L/ATG5 (catalog #ab228668, Abcam, Cambridge, UK), rabbit anti-Beclin1 (catalog #ab62557, Abcam, Cambridge, UK), mouse anti-WT-1 (catalog #ab89901, Abcam, Cambridge, UK), rabbit P-ULK1 (catalog #12753, Cell Signaling Technology, Danvers, MA, USA), ULK (catalog #8054, Cell Signaling Technology, Danvers, MA, USA), p62/SQSTM1 (catalog #ab56416, Abcam, Cambridge, UK), and beta-actin (catalog #ab8227, Abcam, Cambridge, UK).

Total RNA was isolated using TRIzol reagent (catalog #15596026, Life Technologies, Carlsbad, CA, USA) from 30–50 mg of kidney tissue. Additionally, cDNA synthesis and gene expression analysis were performed as previously described (Al-Araimi et al., 2021). In brief, quantitative real-time PCR was performed using SYBR Green Master Mix (Thermo Fisher Scientific, Waltham, MA, USA). Relative gene expression levels were calculated using the relative standard method. The highest standard was made from the cDNA pool, and then, dilutions were made in 1/4 to generate a linear line. Relative quantity for each gene expression was made from the linear equation. Primers were purchased from Metabion ® using salt-out purification, and the sequences are listed in Table 1. Original uncropped Western blot images are included in Supplementary Figure 1.

**TABLE 1:** List of primer sequences used for quantitative PCR.

Gene	Forward primer	Reverse primer
*Atg5*	TGT​GCT​TCG​AGA​TGT​GTG​GTT	GTC​AAA​TAG​CTG​ACT​CTT​GGC​AA
*Becn1*	ATG​GAG​GGG​TCT​AAG​GCG​TC	TCC​TCT​CCT​GAG​TTA​GCC​TCT
*Atg12*	TCC​CCG​GAA​CGA​GGA​ACT​C	TTC​GCT​CCA​CAG​CCC​ATT​TC
*Ulk1*	ACCATTGTCTACCAGTGT	AGT​GTC​TTG​TTC​TTC​TCA​TAA
*Sqstm1*	AGG​ATG​GGG​ACT​TGG​TTG​C	TCA​CAG​ATC​ACA​TTG​GGG​TGC
*b-Actin*	GAT​GTA​TGA​AGG​CTT​TGG​TC	TGT​GCA​CTT​TTA​TTG​GTC​TC
*Fn1*	GAT​GTC​CGA​ACA​GCT​ATT​TAC​CA	CCT​TGC​GAC​TTC​AGC​CAC​T
*Nphs1*	GTG​CCC​TGA​AGG​ACC​CTA​CT	CCT​GTG​GAT​CCC​TTT​GAC​AT
*Nphs2*	CTT​GGC​ACA​TCG​ATC​CCT​CA	CGC​ACT​TTG​GCC​TGT​CTT​TG
*Synpo*	CTT​TGG​GGA​AGA​GGC​CGA​TTG	GTT​TTC​GGT​GAA​GCT​TGT​GC
*Wt1*	GAG​AGC​CAG​CCT​ACC​ATC​C	GGG​TCC​TCG​TGT​TTG​AAG​GAA
*Nos2*	CAT​CAA​CCA​GTA​TTA​TGG​CTC	TTT​CCT​TTG​TTA​CAG​CTT​CC
*Il-1β*	GGA​TGA​TGA​TGA​TAA​CCT​GC	CAT​GGA​GAA​TAT​CAC​TTG​TTG​G
*Tgfβ1*	CAC​CGG​AGA​GCC​CTG​GAT​A	TGT​ACA​GCT​GCC​GCA​CAC​A
*Col1a1*	TTC​ACC​TAC​AGC​ACG​CTT​GTG	GAT​GAC​TGT​CTT​GCC​CCA​AGT​T

### Statistical analysis

The significant differences in quantitative data between control and experimental groups were calculated using GraphPad Prism software v.10 (GraphPad Software, San Diego, CA, USA). Results are expressed as mean ± standard deviation (SD). The Kruskal–Wallis test was performed to compare groups, followed by Tukey’s multiple comparison *post hoc* test to determine statistical differences between them. Group comparison was made between Tre- versus Suc-treated mice within the FSGS− (no disease) or FSGS+ (disease) groups. To compare the effect of Adriamycin within the saline control, we compared between two groups within Tre- or Suc-treated mice.

## Results

### Trehalose ameliorates the markers of subnephrotic proteinuria in a murine FSGS model

We analyzed mice treated with trehalose for 7 weeks after induction of FSGS compared with those treated with sucrose and their respective controls. At the time of sacrifice, mice body weights were not significantly different between the four experimental groups: Suc−, Suc+, Tre−, and Tre+, as shown in Figure 1B. The (−) sign indicates the non-FSGS group and the (+) sign indicates the FSGS Adriamycin-induced group.

At week 7, the degree of renal dysfunction and markers of subnephrotic proteinuria monitored by creatinine clearance, proteinuria changes, albuminuria, and UPCR are shown in Figures 1C–F. In the control group (non-FSGS-induced), trehalose treatment (Tre-) showed no effect on kidney parameters compared to the sucrose control group Suc−, whereas FSGS-induced mice showed significantly lower creatinine clearance (p-value <0.05) than the normal control groups, as shown in Figure 1C. However, no difference was observed in creatinine clearance between the Tre+ and Suc+ groups. We then assessed subnephrotic proteinuria markers and found increased urine protein changes, albuminuria, and UPCR in FSGS-induced mice that received sucrose (Suc− vs. Suc+), as shown in Figures 1D–F. Nevertheless, mice treated with trehalose (Tre+) showed significantly lower albuminuria and UPCR than the Suc+ group (p-value <0.05) with levels comparable to that of the healthy control Tre− group.

### Trehalose reduces kidney fibrosis and podocyte injury in an Adriamycin-induced FSGS model

**FIGURE 2:**
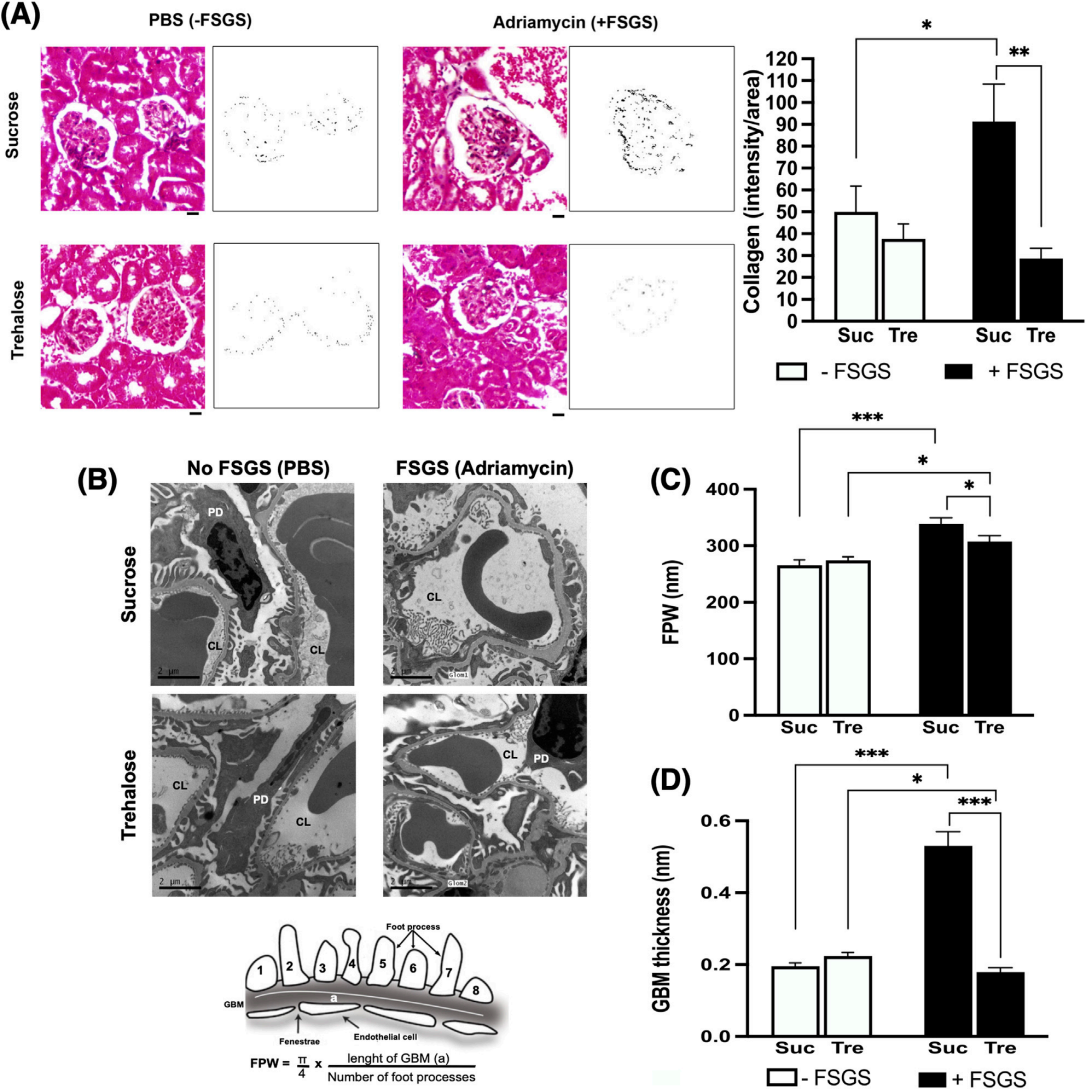
Histopathological analysis of trehalose effect on kidney fibrosis and podocyte integrity. Images are shown at 7 weeks post-administration of trehalose (Tre) or sucrose (Suc) following Adriamycin-induced focal segmental glomerulosclerosis (+FSGS) compared to the control (−FSGS). **(A)** Masson’s trichrome staining of renal glomeruli, with representative images showing color deconvolutions and intensity of collagen deposition (left). At right, collagen deposition was quantified using Fiji software. A 20-μm scale bar is shown below the images using ×40 objective. Images were taken from each mouse in the study (10 mice per group), with a total of 5 glomeruli analyzed per mouse. **(B)** Electron microscopy images of kidney sections showing capillary lumen (CL) and podocytes (PD) with calculation of foot process width (FPW) at bottom. EM was performed on five mice per group. Images were taken from 5 glomeruli of each mouse. **(C,D)** FPW and glomerular basement membrane (GBM) thickness in nm. In GBM analysis, kidney sections from 4–6 mice in each group were analyzed, and measurements were taken in each mouse from 2 or more glomeruli. In each glomerulus, an average thickness was taken from multiple segments in the capillary loop. Tukey’s *post hoc* p-values: *<0.05 and ***<0.001.

Histological assessment of collagen deposition showed marked glomerulosclerosis in sucrose-treated mice after Adriamycin treatment (p-value <0.05), Figure 2A. In contrast, kidney sections from the Tre-treated group showed no increase in collagen deposition and were significantly lower than those in the Suc+ group (p-value<0.05), Figure 2A.

We then assessed podocyte injury using transmission electron microscopy (Figure 2B). Adriamycin-induced damage to podocytes in the Suc+ control group was evidenced by a significant increase in GBM thickness and pronounced foot effacement, reflected by higher FPW, Figures 2C,D. In contrast, trehalose treatment protected against Adriamycin-induced podocyte injury, showing significantly lower GBM thickness and reduced FPW (p-value<0.05).

### Trehalose-protective effects are mediated via enhanced podocyte survival

**FIGURE 3:**
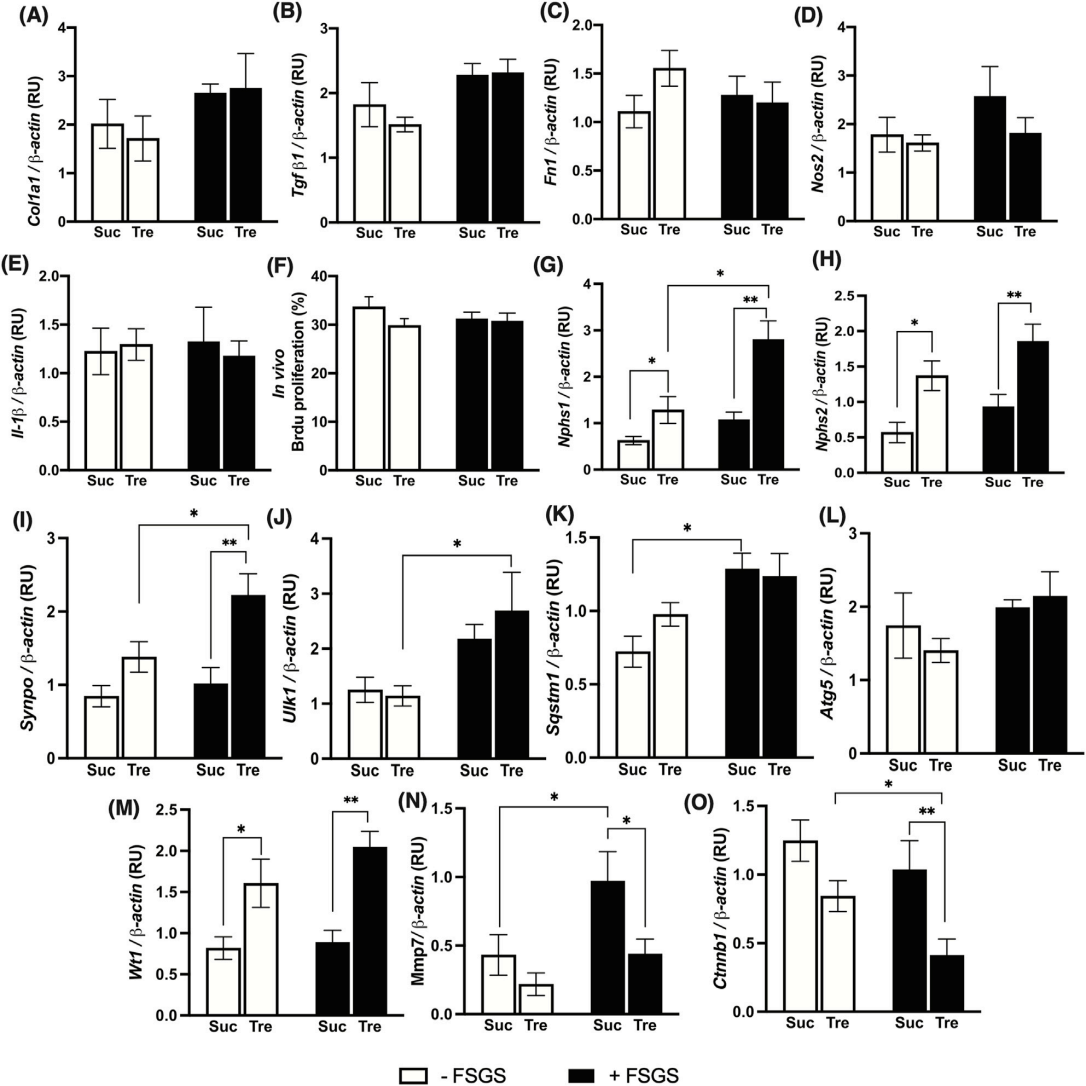
Effect of trehalose administration on markers of fibrosis, proinflammation, and podocytes in an FSGS animal model. FSGS was induced by a single dose of Adriamycin (+) compared to saline (−). Data are shown at the time of sacrifice, 7 weeks post-treatment with trehalose (Tre), versus sucrose (Suc) as the control. **(A–C)** Expression of kidney fibrosis markers. **(D,E)** Expression of pro-inflammatory genes. **(F)** Percentage of bromodeoxyuridine (Brdu)-stained nuclei to total nuclei in renal glomeruli. **(G–I)** Gene expression levels of mature podocyte markers. **(J–L)** Gene expression of autophagy markers. **(M–O)** Expression of Wilms tumor-1 (WT-1) signaling. Gene expressions were normalized to the expression of β-*actin*. The sample size for each group was 10. **RU,** relative unit. Tukey’s *post hoc* p-values: *<0.05, **<0.01, and ***<0.001.

To functionally explain the protective effects of trehalose on albuminuria and histological features of FSGS, we measured the gene expression patterns of different pathways, along with glomerular cellular proliferation. Interestingly, quantitative real-time PCR showed that histological fibrotic changes in the study model were not associated with altered gene expression of pro-fibrotic markers: Procollagen I *(Col1a1), Tgf-β1*, and *Fn1* (Figures 3A–C). Furthermore, there were no changes in pro-inflammatory nitric oxide synthase (*Nos2*) and *Il-1β* gene expressions, Figures 3D,E.

We then examined *in vivo* cellular proliferation within the glomeruli using 3-h Brdu injection prior to sacrifice; however, no significant differences were observed in cellular proliferations within glomeruli of Tre+ and Suc+ groups, Figure 3F. However, significantly higher levels of podocyte markers nephrin 1 (*Nphs1*) and 2 (*Nphs2*), along with synaptopodin (*Synpo*), were detected in Tre+ mice compared to Suc+ littermates in both disease and control groups, Figures 3G–I, with the expressions of these markers being significantly higher in the Tre+ FSGS-induced group, p-value <0.001. This shows increased podocyte integrity through a mechanism independent of inflammation, fibrosis, or proliferation pathways, instead promoting podocyte survival.

### Increased podocyte survival after trehalose administration is mediated via the WT-1 pathway rather than autophagy

**FIGURE 4:**
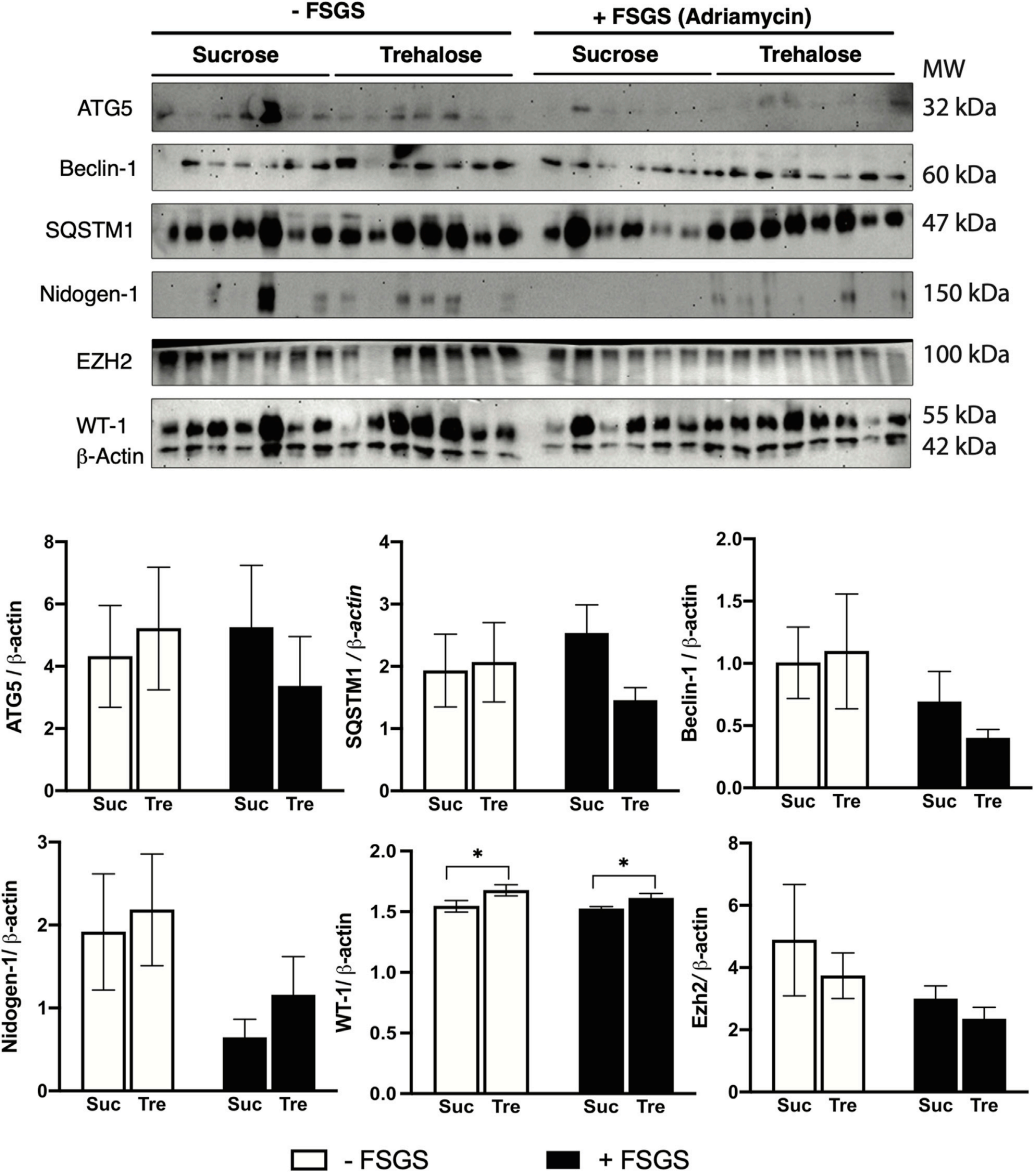
Western blot analysis of trehalose effects in an animal model of FSGS. FSGS was induced by a single dose of Adriamycin (+) compared to saline (−). Data are shown at the time of sacrifice, 7 weeks post-treatment with trehalose (Tre), versus sucrose (Suc) as the control. Western blotting results for ATG5, Beclin-1, SQSTM1, Nidogen-1, EZH-1, and WT-1, along with β-*actin* as a housekeeping control, are shown. A total of seven samples of each group was loaded in one membrane. Bar diagrams indicate quantified densitometry of protein bands normalized to the levels of β-*actin*. Tukey’s *post hoc* p-values: *<0.05.

As mentioned in the introduction, earlier *in vitro* experiments demonstrated that the protective effect of trehalose on podocyte is mediated by the induction of autophagy (Kang et al., 2014). Thus, we herein examined autophagy markers across all experimental groups using Western blotting. Interestingly, there is a significant increase in gene expression patterns of autophagy markers *Ulk1*, *Sqstm1*, and *Atg5* (only trehalose-treated group) in mice with FSGS compared to their healthy counterparts, Figures 3J–L. However, no significant changes were observed between Tre+ and Suc+ groups, except for *Sqstm1* in the non-FSGS control group. Additionally, analysis of renal protein levels also shows no significant differences in ATG5, SQSTM1, and Beclin-1 protein levels in the four experimental groups, Figure 4. These autophagic markers were lower in the Tre+ group than in the Suc+ group but did not reach statistically significant levels.

We then investigated other mechanisms that regulate podocyte health and integrity, namely, nidogen-1 and Wilms tumor suppression protein 1 (WT-1). Our findings indicate that although nidogen-1, a basement membrane marker, is significantly lower in FSGS groups, its protein levels were higher in Tre+ mice, Figure 4. On the other hand, WT-1, an essential marker for the development and homeostasis of podocytes that is frequently measured in kidney biopsies (Guo et al., 2002; Su et al., 2010), increased in renal tissues of Tre+ mice, Figure 3M. This effect was observed in Tre+ mice with and without FSGS, suggesting that it may occur at the transcriptional level. It is worth noting that these results were confirmed at the protein level in Tre+ mice, as shown in Figure 4.

### Trehalose WT-1-enhancing effect is associated with the altered downstream signaling pathway

To further elucidate the mechanism behind the increase in WT-1 signaling, we explored downstream targets of WT-1 at gene and protein expression levels. It has been reported previously that MMP-7 is downregulated in WT-1 knockout mice (Li et al., 2015). Correspondingly, we found reduced expression of *Mmp*-7 in Tre+ mice, which was significantly lower in FSGS mice, Figure 3N. Additionally, previous studies show crosstalk between WT-1 and β-catenin as a protective element of podocyte against diabetic nephropathy via the repression of EZH2/β-catenin (Wagstaff et al., 2023; Corbin et al., 2009; Wan et al., 2017). We herein found that the renal expression of the Catenin β1 gene (*Ctnnb1)* is significantly downregulated in Tre+ mice, with lower levels expressed in the FSGS-induced group, Figure 3O. This is further confirmed by the protein expression levels of EZH2, which are lower in mice treated with trehalose.

## Discussion

This investigation generally aims to elucidate the role of trehalose as a potential therapeutic agent against FSGS. It has been previously reported that Adriamycin can inflect tubulointerstitial injury by altering the glomerular barrier. More specifically, Adriamycin causes direct damage to glomerular endothelial cells, its basement membrane, and podocytes by decreasing their charge density and size selectivity (Lee and Harris, 2011; Jeansson et al., 2009). Thus, Adriamycin was used to induce FSGS in BALB/c mice to investigate the effect of trehalose on the kidney function *in vivo*. Our results indicated that trehalose administration improves proteinuria in mice with FSGS while reducing fibrosis and podocyte damage. This effect is mediated by increased WT-1 signaling, a key pathway to maintain normal podocyte function and growth. Additionally, our findings demonstrate that trehalose can preserve GBM integrity, thereby preventing the significant protein loss observed in the FSGS control group.

One of the major hallmarks of FSGS is the obstruction of glomerular capillaries and pathological fibrosis defined by exaggerated deposition of the extracellular matrix (ECM). In fact, fibrosis is considered a secondary outcome of any severe or progressive glomerular injury or rare disease affecting the podocytes (podocytopathies) (Duffield, 2014). In our model, we observed significant glomerular fibrosis with FSGS-induced mice, which was ameliorated by trehalose treatment. The observation, however, was not associated with changes in gene expression patterns of fibrosis and inflammatory markers at the time of sacrifice. The effect of trehalose may not be involved in the alteration of fibrotic or inflammatory pathways but may improve podocyte health and integrity, resulting in lower fibrosis as shown by histology.

Based on previous *in vitro* investigations, the most significant role of trehalose in FSGS is enhancing the autophagic response, which is essential for podocyte health and function. The association between trehalose and enhanced autophagy has also been demonstrated in other terminally differentiated cells, such as neurons—an effect confirmed by the upregulation of the autophagy markers LC3 and Beclin-1 via the AMPK–ULK1 pathway (Li et al., 2021). Additionally, trehalose appears to suppress inflammatory cytokines by activating the autophagic flux, including the transcription factor EB (TFEB), and by upregulating *Beclin*-1 and *Atg5*/7, thereby forming autophagosomes in corneal epithelial cells (Liu et al., 2020). Moreover, Qu et al. (2019) demonstrated the antioxidative capacity of trehalose using a rat model. This important role is associated with the upregulation of nuclear factor erythroid 2 (Nrf2) via the inhibition of its nuclear translocation and decreasing the expressions of p62/SQSTM1 and LC-311, thereby restoring autophagy (Qu et al., 2019). These findings were confirmed by Tang et al. (2019), who also attributed the reduction in Bcl2, a known apoptosis biomarker, to the effect of trehalose. However, to the best of our knowledge, this is the first investigation on the role of trehalose in preserving podocyte integrity *in vivo*. Our data indicate that although there was a significant increase in gene expression patterns of autophagy markers *Ulk1*, *Sqstm1*, and *Atg5* in mice with FSGS compared to their healthy control, no significant changes were observed between Tre+ and the matched control Suc+. This suggests that the podocyte-protective effects of trehalose under disease conditions could be mediated via factors or pathway other than autophagy.

Our data demonstrate higher markers of mature podocyte at gene and protein expression levels with trehalose treatment. Given that podocyte injury or loss is a common feature in end-stage kidney disease, particularly FSGS, any damage or loss of podocytes presents an irreversible loss of kidney function, leading to proteinuria and glomerulosclerosis (Zhou et al., 2019). From a molecular standpoint, and to assess the effect of trehalose on podocytes, the expression patterns of nephrin (*Nphs1*) and podocin (*Nphs2*) were analyzed. It is important to note that *Nphs1* and *Nphs2* are essential for maintaining a size-selective barrier that forms the intercellular junction in podocytes. Downregulation of these two proteins is known to be associated with severe proteinuria and sclerotic changes (Otaki et al., 2008; Gebeshuber et al., 2013). Our data reveal that both markers, along with synaptopodin, a marker of mature podocytes, are upregulated in trehalose-treated mice, which was significantly higher in the FSGS group than in the control. There are several pathways implicated in such transformation, such as Wnt/β-catenin and WT-1 pathways. In this regard, upregulated Wnt/β-catenin signaling is associated with podocyte injury and dedifferentiation, leading to GMB disruption and proteinuria induction (Li et al., 2021). In a mouse model of Adriamycin-induced nephropathy, activation of Wnt/β-catenin signaling precedes the onset of proteinuria (Dai et al., 2009; Zhou et al., 2019). Similarly, in healthy mice, β-catenin activation through genetic or pharmacological means is sufficient to compromise podocyte integrity and result in proteinuria (Dai et al., 2009; Zhou et al., 2019). On the other hand, the role of WT-1/EZH2/β-catenin was earlier confirmed with regard to podocyte integrity in a diabetic nephropathy model (Wan et al., 2017). More specifically, WT-1, a transcription factor that maintains podocyte homeostasis, reduces the expression of EZH2 at the promotor level and inhibits podocyte damage. Thus, loss of WT-1 downregulates proteins such as podocalyxin and nephrin, leading to the collapse of podocyte architecture (Li et al., 2021). This is significant as EZH2 has been shown to upregulate β-catenin in an osteoarthritis model, causing increased collagen deposition (Chen et al., 2016). However, in the kidney, EZH2 is thought to increase oxidative stress that eventually leads to podocyte death (Wan et al., 2017).

Studying the effect of trehalose on reducing FSGS in mice has its limitation. The short lifespan and controlled laboratory environment of mice do not reflect the complexity of human exposures, comorbidities, and disease progression. Nevertheless, these beneficial effects of trehalose require further exploration in other disease models of podocyte pathology such as diabetic nephropathy before being tested in clinical settings.

Taken together, our findings and previous *in vitro* experiments showed that trehalose is a safe food ingredient that can exert protective effects on podocytes and improve proteinuria in an *in vivo* model of FSGS. Moreover, our study demonstrates that this effect of trehalose is mediated by an upregulation of WT-1 signaling and the concomitant reduction in β-catenin pathways, thus providing mechanistic insights into the protective effect of trehalose on podocyte health and function.

## Data Availability

The original contributions presented in the study are included in the article/Supplementary Material; further inquiries can be directed to the corresponding author.

## References

[B1] Agrawal S. He J. C. Tharaux P. L. (2021). Nuclear receptors in podocyte biology and glomerular disease. Nat. Rev. Nephrol. 17, 185–204. 10.1038/s41581-020-00339-6 32943753

[B2] Al-Araimi A. Al Kindi I. A. Bani Oraba A. AlKharusi A. Ali B. H. Zadjali R. (2021). Gum Arabic supplementation suppresses colonic fibrosis after acute colitis by reducing transforming growth factor *β*1 expression. J. Med. Food 24, 1255–1263. 10.1089/jmf.2021.0007 34704833

[B3] Caster D. J. Magalhaes B. Pennese N. Zaffalon A. Faiella M. Campbell K. N. (2022). Efficacy and safety of immunosuppressive therapy in primary focal segmental glomerulosclerosis: a systematic review and meta-analysis. Kidney Med. 4, 100501. 10.1016/j.xkme.2022.100501 36032548 PMC9399559

[B4] Chen L. Wu Y. Wu Y. Wang Y. Sun L. Li F. (2016). The inhibition of EZH2 ameliorates osteoarthritis development through the Wnt/β-catenin pathway. Sci. Rep. 6, 29176. 10.1038/srep29176 27539752 PMC4990905

[B5] Corbin M. de Reyniès A. Rickman D. S. Berrebi D. Boccon-Gibod L. Cohen-Gogo S. (2009). WNT/beta-catenin pathway activation in wilms tumors: a unifying mechanism with multiple entries? Genes Chromosom. Cancer 48, 816–827. 10.1002/gcc.20686 19530245

[B6] Dai C. Stolz D. B. Kiss L. P. Monga S. P. Holzman L. B. Liu Y. (2009). Wnt/beta-Catenin signaling promotes podocyte dysfunction and albuminuria. J. Am. Soc. Nephrol. 20, 1997–2008. 10.1681/ASN.2009010019 19628668 PMC2736766

[B7] Darabi S. Noori-Zadeh A. Abbaszadeh H. A. Rajaei F. (2018). Trehalose activates autophagy and prevents Hydrogen peroxide-induced apoptosis in the bone marrow stromal cells. Iran. J. Pharm. Res. 17, 1141–1149. 10.22037/ijpr.2018.2277 30127837 PMC6094425

[B8] Duffield J. S. (2014). Cellular and molecular mechanisms in kidney fibrosis. J. Clin. Invest 124, 2299–2306. 10.1172/JCI72267 24892703 PMC4038570

[B9] Galbiati M. Meroni M. Boido M. Cescon M. Rusmini P. Crippa V. (2023). Bicalutamide and trehalose ameliorate spinal and bulbar muscular atrophy pathology in mice. Neurotherapeutics 20, 524–545. 10.1007/s13311-023-01343-x 36717478 PMC10121997

[B10] Gebeshuber C. A. Kornauth C. Dong L. Sierig R. Seibler J. Reiss M. (2013). Focal segmental glomerulosclerosis is induced by microRNA-193a and its downregulation of WT1. Nat. Med. 19, 481–487. 10.1038/nm.3142 23502960

[B11] Guo J. K. Menke A. L. Gubler M. C. Clarke A. R. Harrison D. Hammes A. (2002). WT1 is a key regulator of podocyte function: reduced expression levels cause crescentic glomerulonephritis and mesangial sclerosis. Hum. Mol. Genet. 11, 651–659. 10.1093/hmg/11.6.651 11912180

[B12] Hartleben B. Wanner N. Huber T. B. (2014). Autophagy in glomerular health and disease. Semin. Nephrol. 34, 42–52. 10.1016/j.semnephrol.2013.11.007 24485029

[B13] Hom M. M. Ladhani O. Zhang Z. Liu H. Sonparote S. Dancey C. C. (2025). Patient experience with ABBV-444, a proof-of-concept Study for a novel artificial tear with trehalose and sodium hyaluronate for dry eye symptoms. Clin. Optom. (Auckl) 17, 37–45. 10.2147/OPTO.S490732 39963316 PMC11830937

[B14] Hosseinpour-Moghaddam K. Caraglia M. Sahebkar A. (2018). Autophagy induction by trehalose: molecular mechanisms and therapeutic impacts. J. Cell Physiol. 233, 6524–6543. 10.1002/jcp.26583 29663416 PMC13482534

[B15] Jacobs-Cacha C. Vergara A. García-Carro C. Agraz I. Toapanta-Gaibor N. Ariceta G. (2021). Challenges in primary focal segmental glomerulosclerosis diagnosis: from the diagnostic algorithm to novel biomarkers. Clin. Kidney J. 14, 482–491. 10.1093/ckj/sfaa110 33623672 PMC7886539

[B16] Jeansson M. Bjorck K. Tenstad O. Haraldsson B. (2009). Adriamycin alters glomerular endothelium to induce proteinuria. J. Am. Soc. Nephrol. 20, 114–122. 10.1681/ASN.2007111205 19073829 PMC2615716

[B17] Joles J. A. van Goor H. Koomans H. A. (1998). Estrogen induces glomerulosclerosis in analbuminemic rats. Kidney Int. 53, 862–868. 10.1111/j.1523-1755.1998.00825.x 9551392

[B18] Kang Y. L. Saleem M. A. Chan K. W. Yung B. Y. Law H. K. (2014). Trehalose, an mTOR independent autophagy inducer, alleviates human podocyte injury after puromycin aminonucleoside treatment. PLoS One 9, e113520. 10.1371/journal.pone.0113520 25412249 PMC4239098

[B19] Latt K. Z. Heymann J. Jessee J. H. Rosenberg A. Z. Berthier C. C. Arazi A. (2022). Urine single-cell RNA sequencing in focal segmental glomerulosclerosis reveals inflammatory signatures. Kidney Int. Rep. 7, 289–304. 10.1016/j.ekir.2021.11.005 35155868 PMC8821042

[B20] Lee V. W. Harris D. C. (2011). Adriamycin nephropathy: a model of focal segmental glomerulosclerosis. Nephrol. Carlt. 16, 30–38. 10.1111/j.1440-1797.2010.01383.x 21175974

[B21] Li X. Ottosson S. Wang S. Jernberg E. Boldrup L. Gu X. (2015). Wilms' tumor gene 1 regulates p63 and promotes cell proliferation in squamous cell carcinoma of the head and neck. BMC Cancer 15, 342. 10.1186/s12885-015-1356-0 25929687 PMC4421988

[B22] Li T. Yu C. Zhuang S. (2021). Histone methyltransferase EZH2: a potential therapeutic target for kidney diseases. Front. Physiol. 12, 640700. 10.3389/fphys.2021.640700 33679454 PMC7930071

[B23] Liu Z. Chen D. Chen X. Bian F. Qin W. Gao N. (2020). Trehalose induces autophagy against inflammation by activating TFEB signaling pathway in human corneal epithelial cells exposed to hyperosmotic stress. Invest Ophthalmol. Vis. Sci. 61, 26. 10.1167/iovs.61.10.26 32785678 PMC7441355

[B24] Musiala A. Donizy P. Augustyniak-Bartosik H. Jakuszko K. Banasik M. Kościelska-Kasprzak K. (2022). Biomarkers in primary focal segmental glomerulosclerosis in optimal diagnostic-therapeutic strategy. J. Clin. Med. 11, 3292. 10.3390/jcm11123292 35743361 PMC9225193

[B25] Otaki Y. Miyauchi N. Higa M. Takada A. Kuroda T. Gejyo F. (2008). Dissociation of NEPH1 from nephrin is involved in development of a rat model of focal segmental glomerulosclerosis. Am. J. Physiol. Ren. Physiol. 295, F1376–F1387. 10.1152/ajprenal.00075.2008 18715943

[B26] Qu K. C. Wang Z. Y. Tang K. K. Zhu Y. S. Fan R. F. (2019). Trehalose suppresses cadmium-activated Nrf2 signaling pathway to protect against spleen injury. Ecotoxicol. Environ. Saf. 181, 224–230. 10.1016/j.ecoenv.2019.06.007 31195231

[B27] Ruby M. Gifford C. C. Pandey R. Raj V. S. Sabbisetti V. S. Ajay A. K. (2023). Autophagy as a therapeutic target for chronic kidney disease and the roles of TGF-β1 in autophagy and kidney fibrosis. Cells 12, 412. 10.3390/cells12030412 36766754 PMC9913737

[B28] Santmyire B. R. Venkat V. Beinder E. Baylis C. (2010). Impact of the estrus cycle and reduction in estrogen levels with aromatase inhibition, on renal function and nitric oxide activity in female rats. Steroids 75, 1011–1015. 10.1016/j.steroids.2010.06.016 20619284 PMC2926238

[B29] Savin V. J. Sharma R. Sharma M. McCarthy E. T. Swan S. K. Ellis E. (1996). Circulating factor associated with increased glomerular permeability to albumin in recurrent focal segmental glomerulosclerosis. N. Engl. J. Med. 334, 878–883. 10.1056/NEJM199604043341402 8596570

[B30] Schindelin J. Arganda-Carreras I. Frise E. Kaynig V. Longair M. Pietzsch T. (2012). Fiji: an open-source platform for biological-image analysis. Nat. Methods 9, 676–682. 10.1038/nmeth.2019 22743772 PMC3855844

[B31] Shabaka A. Tato Ribera A. Fernandez-Juarez G. (2020). Focal segmental glomerulosclerosis: State-of-the-Art and clinical perspective. Nephron 144, 413–427. 10.1159/000508099 32721952

[B32] Su J. Li S. J. Chen Z. H. Zeng C. H. Zhou H. Li L. S. (2010). Evaluation of podocyte lesion in patients with diabetic nephropathy: wilms' tumor-1 protein used as a podocyte marker. Diabetes Res. Clin. Pract. 87, 167–175. 10.1016/j.diabres.2009.10.022 19969384

[B33] Tang K. K. Liu X. Y. Wang Z. Y. Qu K. C. Fan R. F. (2019). Trehalose alleviates cadmium-induced brain damage by ameliorating oxidative stress, autophagy inhibition, and apoptosis. Metallomics 11, 2043–2051. 10.1039/c9mt00227h 31650140

[B34] Trachtman H. (2020). Emerging drugs for treatment of focal segmental glomerulosclerosis. Expert Opin. Emerg. Drugs 25, 367–375. 10.1080/14728214.2020.1803276 32729368 PMC7508791

[B35] Trial H. A. P. Group H. A. P. T. S. (2025). Safety and efficacy of trehalose in amyotrophic lateral sclerosis (HEALEY ALS Platform Trial): an adaptive, phase 2/3, double-blind, randomised, placebo-controlled trial. Lancet Neurol. 24, 500–511. 10.1016/S1474-4422(25)00173-5 40409314

[B36] van Can J. G. van Loon L. J. Brouns F. Blaak E. E. (2012). Reduced glycaemic and insulinaemic responses following trehalose and isomaltulose ingestion: implications for postprandial substrate use in impaired glucose-tolerant subjects. Br. J. Nutr. 108, 1210–1217. 10.1017/S0007114511006714 22172468

[B37] van den Berg J. G. van den Bergh Weerman M. A. Assmann K. J. Weening J. J. Florquin S. (2004). Podocyte foot process effacement is not correlated with the level of proteinuria in human glomerulopathies. Kidney Int. 66, 1901–1906. 10.1111/j.1523-1755.2004.00964.x 15496161

[B38] Wagstaff M. Tsaponina O. Caalim G. Greenfield H. Milton-Harris L. Mancini E. J. (2023). Crosstalk between beta-catenin and WT1 signaling activity in acute myeloid leukemia. Haematologica 108, 283–289. 10.3324/haematol.2021.280294 35443562 PMC9827145

[B39] Wan J. Hou X. Zhou Z. Geng J. Tian J. Bai X. (2017). WT1 ameliorates podocyte injury *via* repression of EZH2/β-catenin pathway in diabetic nephropathy. Free Radic. Biol. Med. 108, 280–299. 10.1016/j.freeradbiomed.2017.03.012 28315733

[B40] Wen Y. Shah S. Campbell K. N. (2018). Molecular mechanisms of proteinuria in focal segmental glomerulosclerosis. Front. Med. (Lausanne) 5, 98. 10.3389/fmed.2018.00098 29713631 PMC5912003

[B41] Wyburn K. R. Chadban S. J. Kwan T. Alexander S. I. Wu H. (2013). Interleukin-18 binding protein therapy is protective in adriamycin nephropathy. Am. J. Physiol. Ren. Physiol. 304, F68–F76. 10.1152/ajprenal.00669.2011 23097468

[B42] Xu C. Liu X. Zhai X. Wang G. Qin W. Cheng Z. (2023). CDDO-Me ameliorates podocyte injury through anti-oxidative stress and regulation of actin cytoskeleton in adriamycin nephropathy. Biomed. Pharmacother. 167, 115617. 10.1016/j.biopha.2023.115617 37801905

[B43] Yi M. Zhang L. Liu Y. Livingston M. J. Chen J. K. Nahman N. S. Jr (2017). Autophagy is activated to protect against podocyte injury in adriamycin-induced nephropathy. Am. J. Physiol. Ren. Physiol. 313, F74–F84. 10.1152/ajprenal.00114.2017 28404589 PMC5538842

[B44] Yuan Q. Tang B. Zhang C. (2022). Signaling pathways of chronic kidney diseases, implications for therapeutics. Signal Transduct. Target Ther. 7, 182. 10.1038/s41392-022-01036-5 35680856 PMC9184651

[B45] Zadjali F. Santana-Farre R. Vesterlund M. Carow B. Mirecki-Garrido M. Hernandez-Hernandez I. (2012). SOCS2 deletion protects against hepatic steatosis but worsens insulin resistance in high-fat-diet-fed mice. FASEB J. 26, 3282–3291. 10.1096/fj.12-205583 22562833

[B46] Zhou L. Chen X. Lu M. Wu Q. Yuan Q. Hu C. (2019). Wnt/β-catenin links oxidative stress to podocyte injury and proteinuria. Kidney Int. 95, 830–845. 10.1016/j.kint.2018.10.032 30770219 PMC6431566

[B47] Zhu X. Tang L. Mao J. Hameed Y. Zhang J. Li N. (2022). Decoding the mechanism behind the pathogenesis of the focal segmental glomerulosclerosis. Comput. Math. Methods Med. 2022, 1941038. 10.1155/2022/1941038 35693262 PMC9175094

[B48] Zschiedrich S. Bork T. Liang W. Wanner N. Eulenbruch K. Munder S. (2017). Targeting mTOR signaling can prevent the progression of FSGS. J. Am. Soc. Nephrol. 28, 2144–2157. 10.1681/ASN.2016050519 28270414 PMC5491276

