# Supplementary figures and images for: Trehalose alleviates nephropathy in focal segmental glomerulosclerosis via the upregulation of the WT-1/EZH2 pathway
Source: Front Pharmacol. 2025 Nov 6;16:1706617. doi: 10.3389/fphar.2025.1706617 (PMC12630111; doi:10.3389/fphar.2025.1706617)

supplementary figure  
original Western blot images

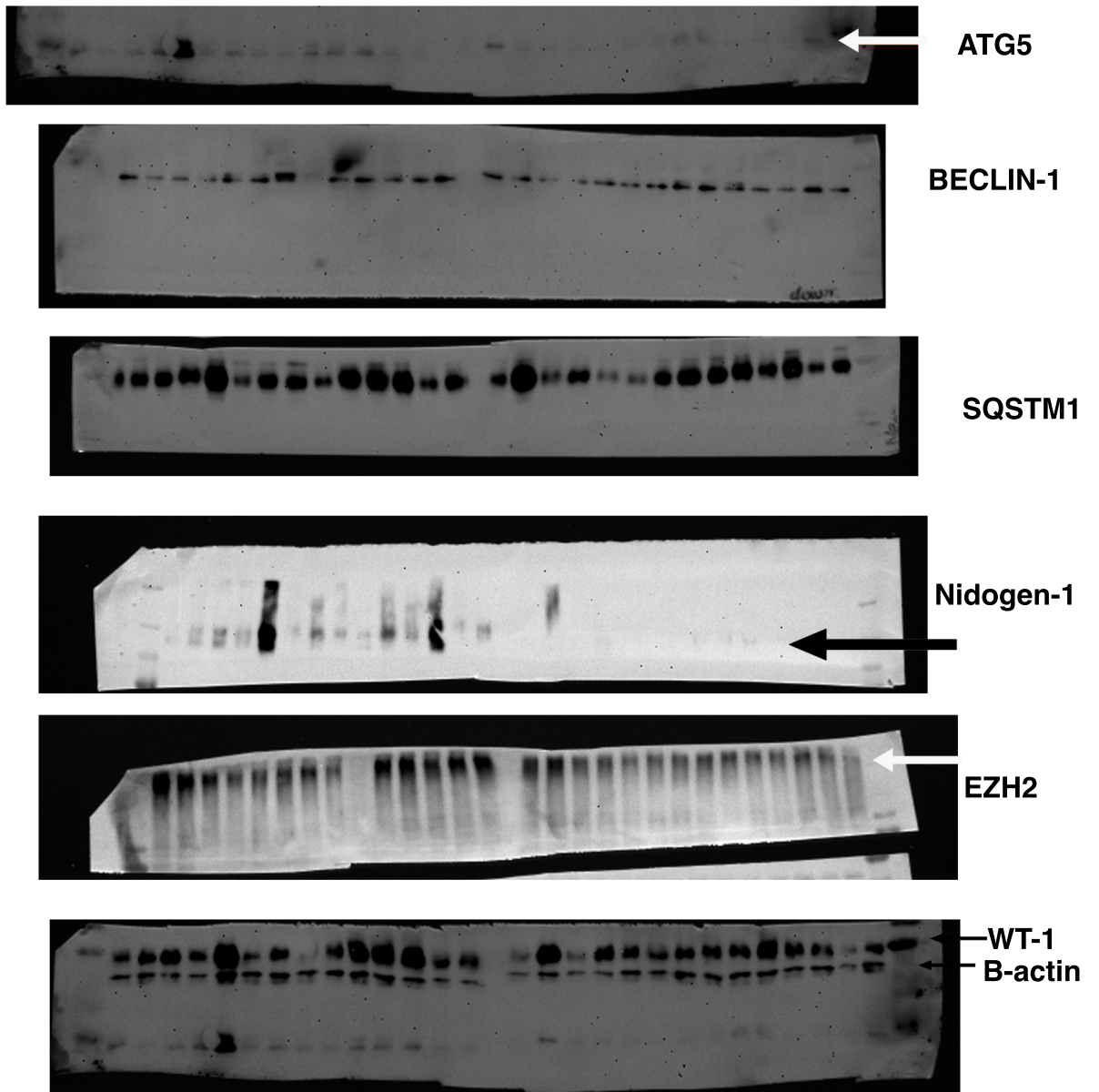

Supplement: Supplementary file 1 [file DataSheet1.pdf]
